# Acarbose has sex-dependent and -independent effects on age-related physical function, cardiac health, and lipid biology

**DOI:** 10.1172/jci.insight.137474

**Published:** 2020-11-05

**Authors:** Jonathan J. Herrera, Sean Louzon, Kaitlyn Pifer, Danielle Leander, Gennifer E. Merrihew, Jea H. Park, Kate Szczesniak, Jeremy Whitson, John E. Wilkinson, Oliver Fiehn, Michael J. MacCoss, Sharlene M. Day, Richard A. Miller, Michael Garratt

**Affiliations:** 1 Department of Molecular & Integrative Physiology, University of Michigan (UM), Ann Arbor, Michigan, USA.; 2 Department of Pathology, UM Medical School, Ann Arbor, Michigan, USA.; 3 Department of Genome Sciences and; 4 Department of Pathology, University of Washington, Seattle, Washington, USA.; 5 Unit for Laboratory Animal Medicine and Department of Pathology, UM, Ann Arbor, Michigan, USA.; 6 UCD, Genome Center, Davis, California, USA.; 7 Department of Medicine, University of Pennsylvania, Philadelphia, Pennsylvania, USA.; 8 UM Geriatrics Center, Ann Arbor, Michigan, USA.; 9 Department of Anatomy, School of Biomedical Sciences, University of Otago, Dunedin, New Zealand.

**Keywords:** Aging, Cellular senescence

## Abstract

With an expanding aging population burdened with comorbidities, there is considerable interest in treatments that optimize health in later life. Acarbose (ACA), a drug used clinically to treat type 2 diabetes mellitus (T2DM), can extend mouse life span with greater effect in males than in females. Using a genetically heterogeneous mouse model, we tested the ability of ACA to ameliorate functional, pathological, and biochemical changes that occur during aging, and we determined which of the effects of age and drug were sex dependent. In both sexes, ACA prevented age-dependent loss of body mass, in addition to improving balance/coordination on an accelerating rotarod, rotarod endurance, and grip strength test. Age-related cardiac hypertrophy was seen only in male mice, and this male-specific aging effect was attenuated by ACA. ACA-sensitive cardiac changes were associated with reduced activation of cardiac growth–promoting pathways and increased abundance of peroxisomal proteins involved in lipid metabolism. ACA further ameliorated age-associated changes in cardiac lipid species, particularly lysophospholipids — changes that have previously been associated with aging, cardiac dysfunction, and cardiovascular disease in humans. In the liver, ACA had pronounced effects on lipid handling in both sexes, reducing hepatic lipidosis during aging and shifting the liver lipidome in adulthood, particularly favoring reduced triglyceride (TAG) accumulation. Our results demonstrate that ACA, already in clinical use for T2DM, has broad-ranging antiaging effects in multiple tissues, and it may have the potential to increase physical function and alter lipid biology to preserve or improve health at older ages.

## Abstract 2

Acarbose treatment ameliorates age-associated declines in physical function and cardiac structural ageing, changes that are linked to slowed changes in the cardiac lipidome.

## Introduction

Because the effects of aging in multiple physiological systems lead to clinically significant comorbidities and deteriorating systemic health, therapies that target the aging process may possess significant clinical value by prevention of physiological decline, concordant with their expected effects on life span.

Several pharmacological interventions have been shown to extend life span in mice. The extension of life span, however, does not always affect both sexes in a similar manner, and a paucity of information exists about the effects of such treatments on tissue-specific indices of health (1). Two drugs approved for human use by the Food and Drug Administration (FDA), rapamycin and acarbose (ACA), have led to significant life span extension in UM-HET3 male and female mice (2–5). Rapamycin, most widely studied, can lead to an increase in life span of ~10%–20% in both sexes and can ameliorate a subset of age-related phenotypic changes in mice (6, 7). However, its side effects, which in humans can include thrombocytopenia, nephrotoxicity, and impaired wound healing (8), may limit its potential as an antiaging therapeutic.

ACA extends life span by ~15% in males but has a smaller (~5%) effect on life span in females. ACA blunts postprandial glucose uptake from the small intestine by inhibiting α-glycosidases and, thus, slows breakdown of complex carbohydrates to absorbable sugars (9). The drug is prescribed clinically as an oral type 2 diabetes mellitus (T2DM) medication that decreases progression from prediabetes to diabetes in humans (10), and it has been shown to improve both glucose (11, 12) and lipid profiles in T2DM patients (13, 14). Preclinical mouse studies have recapitulated these findings and have provided preliminary evidence that ACA treatment may prevent vascular complications and the development of cardiovascular, renal, retinal, metabolic and neurologic complications in the context of diabetes (15).

Delayed onset of T2DM by ACA may reduce the risk of cardiovascular disease and mortality in at-risk populations (14, 16). Side effects of ACA are mild and primarily result in flatulence but can also include abdominal discomfort and loose stools (9). These adverse effects, however, can be mitigated by changes in drug administration and diet (17). In longer-term studies in the absence of diabetes, ACA promoted body weight reductions in rodents (12) and humans (18, 19) and counteracted age-related dysfunction in glucose and insulin regulation in rats (20). These effects on physiological glucose maintenance and insulin sensitivity have been postulated to underlie observed increases in mouse longevity with ACA treatment (2).

Given the beneficial metabolic effects in disease and nondisease states, ACA might be useful, in humans, not only as an antidiabetic agent, but potentially as an intervention to promote healthy aging. Whether ACA preserves systemic health and function with age has not yet been fully investigated. To evaluate the effects of long-term ACA treatment on mouse aging, we tested a series of biometric, functional, and biochemical outcomes to evaluate multiple aspects of health in male and female UM-HET3 mice. UM-HET3 mice are a genetically heterogenous breed of mouse, derived from a 4-way cross of inbred strains. This limits the occurrence of strain-specific idiosyncrasies in pathology (21). We used a dose of ACA (1000 mg per kg diet) that optimizes life span extension in both sexes, with a higher dose (2500 mg/kg diet) failing to increase life span further in either sex (22). This allowed us to test whether ACA has sex-specific effects on aspects of age-associated health, matching life span extension, or whether the treatment effects are accrued equally in both sexes.

## Results

### ACA differentially influences body weight across different life periods.

**Figure 1:**
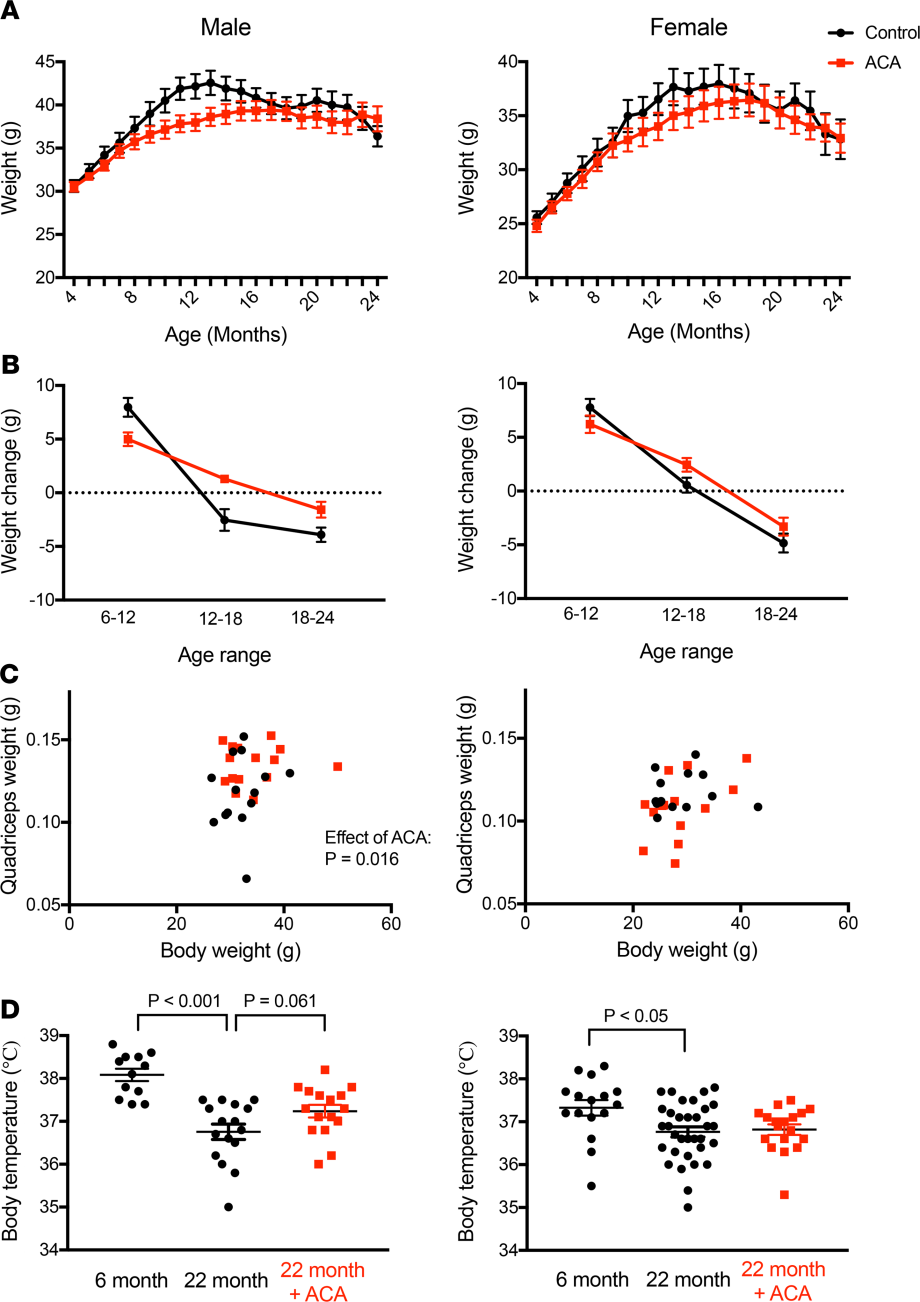
Effects of ACA on body weight dynamics, muscle mass, and body temperature. (**A** and **B**) Body weight was assessed monthly from the initiation of treatment at 4 months until 25 months of age (**A**), and data were used to calculate weight change across different time points (**B**). *n* = 16–19 per sex and treatment group at the start of the study, with 14–16 mice per group remaining alive at 24 months. (**C**) At 25 months, animals were dissected and quadriceps weight was higher in male mice treated with ACA compared with controls. (**D**) In a separate cohort of mice treated with ACA from 4 months, body temperature was assessed at 22 months of age and compared with untreated animals that were of the same age, in addition to younger 6 month old animals. *P* values in **C** were calculated with an ANCOVA, including body weight as a covariate. In **D** and **E**, *P* values are calculated from a Dunnett’s multiple comparisons test, comparing young animals and old ACA-treated animals with old controls in separate comparisons, after conducting an ANOVA. Each dot represents a data point from an individual animal.

Mice treated with ACA from 4 to 25 months of age were weighed monthly to assess changes in body weight over time. Effects of ACA on body weight differed depending on life stage. ACA reduced weight gain early in life but opposed the loss of weight in older mice (Figure 1A). ACA reduces weight gain over the first 12 months of life in both sexes (effect of ACA, *P* = 0.005; ACA × sex interaction, *P* = 0.37). This is the period over which control animals gained weight. In contrast, ACA-treated mice showed significantly less reduction in body weight compared with controls over the 12- to 18-month and 18- to 24-month age intervals (effect of ACA: 12–18 months, *P* < 0.001; 18–24 months, *P* = 0.018) (Figure 1B). These effects were similar in both sexes (ACA × sex interaction: 12–18 months, *P* = 0.16; 18–24 months, *P* = 0.61). When dissected at 25 months, ACA-treated mice did not differ from controls in the weight of mesenteric, inguinal, or gonadal fat pads (Supplemental Data Set 1; supplemental material available online with this article; https://doi.org/10.1172/jci.insight.137474DS1). Quadriceps muscle of ACA-treated male mice was significantly heavier than in controls, suggesting that ACA treatment helps prevent muscle atrophy and contributes to maintenance of body mass during aging in males (Figure 1C). Female quadriceps muscle mass was unaffected by ACA treatment. In a separate cohort, we measured body temperature at 22 months of age; this also declines in mice at later ages and is suggested to represent a loss of homeostatic regulation (23, 24). Body temperature of old control male mice was indeed lower than in young counterparts, and while this decline was also observed in females, a stronger decline in body temperature with age was observed in males than females (Figure 1D; age × sex interaction, *P* = 0.023). ACA treatment did not significantly effect the body temperature of either sex when compared with untreated old controls (Figure 1D).

### ACA improves physical function in both sexes.

**Figure 2:**
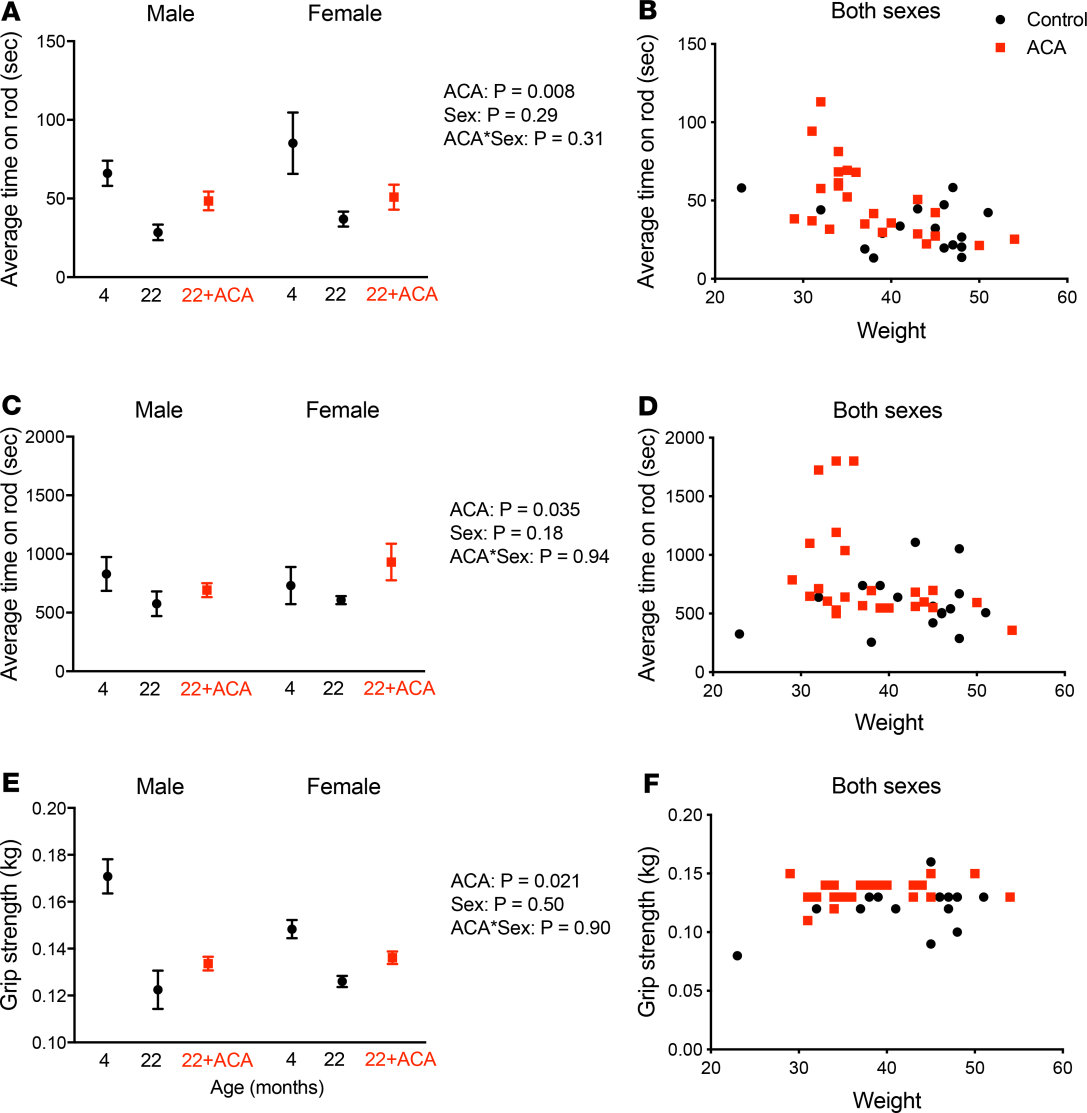
ACA improves physical function in old mice at 22 months of age. Balance capacity was assessed on an accelerating rotarod (**A** and **B**), while endurance capacity was tested by maintaining mice on the rod at a submaximal speed (**C** and **D**). (**A**, **C**, and **E**) Data are presented as mean ± SEM, with data split according to age, sex ,and ACA treatment. (**B**, **D**, and **F**) Individual data for functional capacity of each mouse in relation to body weight (data from both sexes pooled because sex did not influence scores) (*n* = 8–12 per sex for each group). Grip strength was assessed with a grip strength meter (**E** and **F**). *P* values were calculated from a 2-way ANOVA on data from 22 month old mice, including sex, ACA and their interaction as factors.

Young (6 months), old (22 months), and ACA-treated old mice (ACA treatment from 4 months) were subjected to a series of physical function tests to assess balance/coordination capacity, endurance, and strength. We observed age-related declines in these series of tests, but we also found that ACA treatment was protective in both sexes. When evaluating the capacity of mice to remain on an accelerating rotarod over a period of 3–4 minutes, ACA increased fall latency (Figure 2A, P** = 0.008), an effect that was independent of sex (ACA × sex interaction, *P* = 0.31). Because ACA can influence body weight, we also tested whether the effects of ACA on the accelerating rotarod test might reflect overall body weight differences, which were still mildly apparent at 22 months (Figure 1, Figure 2B for the physical function cohort). Rotarod performance was indeed negatively related to body weight (Figure 2B, P** = 0.0001 for effect of weight as a continuous covariate). When this negative relationship is accounted for by including body weight as a covariate, the effect of ACA treatment was no longer significant (*P* = 0.15). This indicates that the effects of ACA on rotarod performance could be attributable, at least in part, to the ACA-associated reduction in body weight. We conducted an additional test of physical function, in which mice were placed on the rotarod at a slower, continuous speed (rotarod endurance test, Figure 2C). Body weight did not predict endurance capacity at 22 months (Figure 2D, P** = 0.19 effect of body weight as a covariate), and mice treated with ACA showed a significant improvement compared with untreated controls in both sexes (Figure 2C) (ACA effect, *P* = 0.035; ACA × sex interaction, *P* = 0.94). We also evaluated forepaw grip strength and observed an age-related decline in grip strength, which was attenuated by ACA treatment in both sexes (*P* = 0.021; ACA × sex interaction, *P* = 0.90) (Figure 2E). Body weight was positively associated with grip strength (*P* = 0.01). When controlling for body weight as a covariate, the effect of ACA was stronger (Figure 2F, P** = 0.002), indicating that ACA mice have a greater grip strength than untreated mice for a given body weight.

### Age-associated changes in cardiac remodeling are reduced by ACA in males.

**Figure 3:**
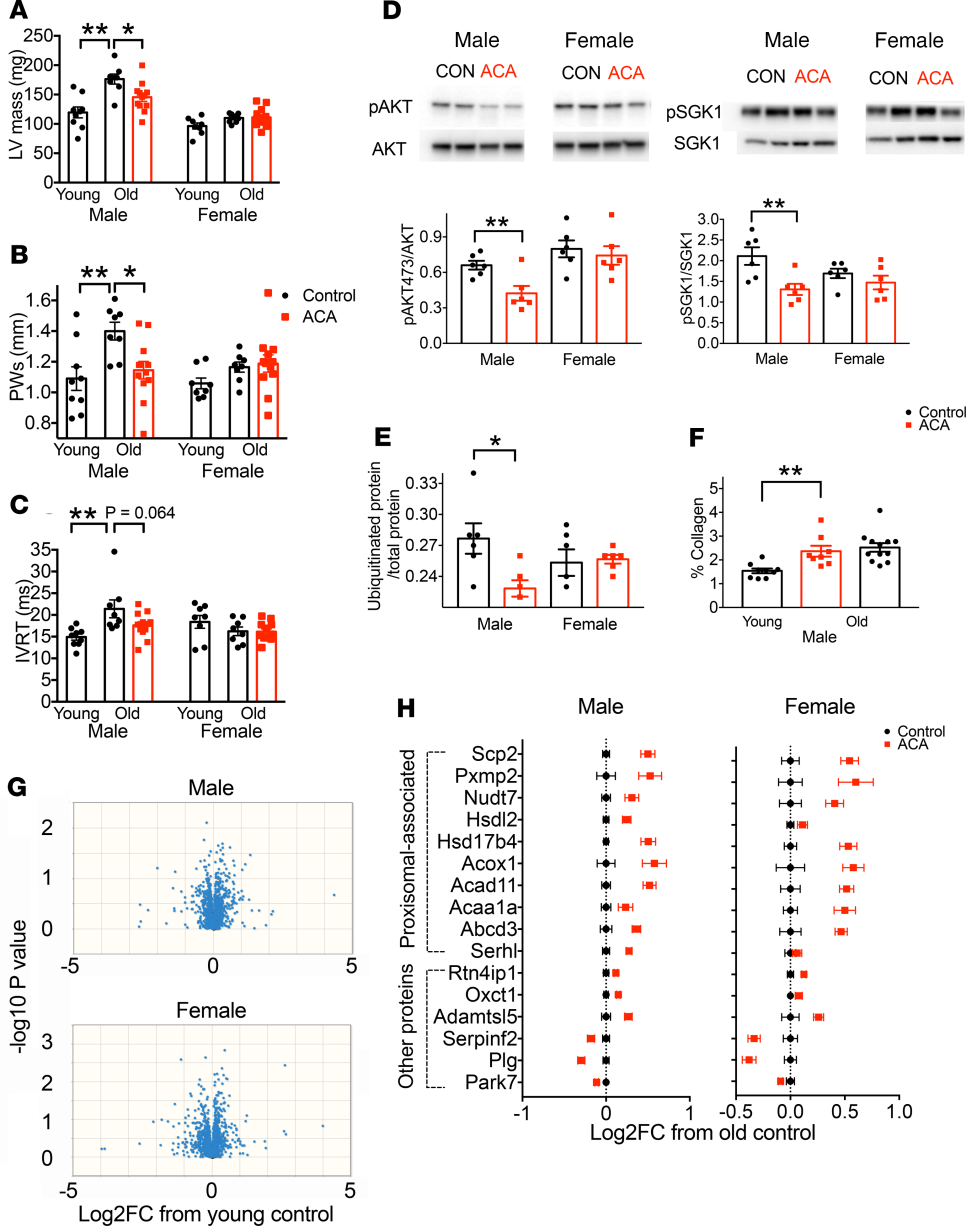
Effect of ACA on heart structure, function, signaling, and proteomic composition. (**A**–**C**) Echocardiography was conducted on mice at 22 months of age (*n* = 8 per sex for old controls; *n* = 12 per sex for ACA treated), and included a set of young (6-month) controls (*n* = 8 per sex). (**D** and **E**) Analysis of cardiac signaling pathways and ubiquitinated protein was conducted on tissue samples from 25-month-old mice by Western blot. (**F**) The relative presence of collagen was assessed by Picrosirius red staining. (**G**) Proteomics analysis was conducted on samples from 25-month-old mice and 6-month-old young controls (*n* = 8 per sex for each treatment group). Volcano plots for each sex are shown, highlighting the lack of proteins that have a consistent change with age in protein abundance. (**H**) The log_2_ fold change in protein abundance from old control animals is shown for old mice treated with ACA for each protein that differs between control and ACA-treated mice after correction for FDR. Each error bar is a value for a different protein, with the figure showing relative change in abundance of proteins differing significantly between young and old mice on a control diet. Proteins were split according to their association with peroxisome, as defined by Gene Ontology (GO) cellular component annotation terms.*P < 0.05, **P < 0.01. In **A**–**C** and **F**, the *P* values were computed by running an ANOVA then Dunnett’s multiple comparison test, comparing young males and old ACA-treated males with old control males in separate comparisons. In **D**, *P* values were calculated with a Student’s *t* test.

To investigate whether ACA influenced cardiac structural remodeling or function during aging, we used echocardiography, comparing the hearts of young, old, and old ACA-treated mice. We observed remodeling in male mice hearts consistent with age-associated cardiac hypertrophy. Male mice show a significant increase in left ventricle mass and posterior wall thickness in systole (PWs) with aging, while female mice tended to show less of an age-associated response (age × sex interactions: left ventricular [LV] mass, *P* = 0.005; PWs, *P* = 0.08) (Figure 3, A and B). ACA reduced male-specific hypertrophy in old animals in a sex-specific manner (ACA × sex interactions: LV mass, *P* = 0.017; PWs, *P* = 0.018), without significant effects in females (Figure 3, A and B). We observed no age effects on measures of diastolic function in either sex, including E/A ratio or myocardial performance index (MPI), which have previously been found to change with age in C57BL/6J mice (25), and there was no effect of ACA on these parameters (Supplemental Tables 1 and 2). Aged male mice did, however, demonstrate a prolonged isovolumetric relaxation time (IVRT) (age × sex interaction, *P* = 0.004; Figure 3C), another index of diastolic function observed in cardiac aging in humans (26) and rodent models (27). ACA treatment did not significantly effect this parameter in old males. There was a trend for this age-related change to lessen in ACA-treated mice, although the difference between old control and old ACA-treated males did not reach statistical significance (*P* = 0.064, Figure 3C), and there was no overall effect of ACA in a 2-way ANOVA, including data from both sexes (*P* = 0.090), nor was there an interaction between sex and ACA treatment (*P* = 0.11).

Cardiac hypertrophy in mice is characterized by chronic activation of growth-promoting signaling pathways downstream of the insulin receptor, with increased activation of AKT and SGK1 contributing to a hypertrophic response in various conditions (28–30). We hypothesized that the effects of ACA on glucose metabolism (15) would reduce the activation status of these proteins during aging. ACA treatment reduced phosphorylation at sites involved in protein activation when assessed at 25 months of age in whole heart samples. In each case, reduced phosphorylation was observed in ACA-treated male mice (Figure 3D; see complete unedited blots in the supplemental material.). However, evidence of a male-specific change with ACA was not observed, as the 2-way ANOVA showed that reduced phosphorylation occurred similarly in both sexes (pSGK1, ACA × sex interaction [*P* = 0.086], treatment effect for both sexes [*P* = 0.005]; pAKT at serine 473, ACA × sex interaction [*P* = 0.17], treatment effect for both sexes [*P* = 0.034]). Impaired proteasomal function has been proposed to also contribute to maladaptive cardiac hypertrophy, with high levels of ubiquitinated proteins a possible indicator for defective cellular protein breakdown (31). We detected a sex-specific change in the accumulation of ubiquitinated protein in the heart at 25 months with ACA treatment (sex × treatment interaction, *P* = 0.026). This was reduced in ACA-treated males but not females (Figure 3E). We also tested whether ACA reduced cardiac fibrosis, a pathologic remodeling phenotype linked to cardiac aging (32). Old male mice showed a clear elevation in fibrosis when compared with young males, assessed by Picrosirius red staining for collagen, but this was unaffected by ACA treatment (Figure 3F).

### ACA increases the abundance of peroxisomal proteins in cardiac tissue.

Previous investigations of slowed cardiac aging with short-term rapamycin treatment and calorie restriction have documented a major change in the cardiac proteome of C57BL/6J female mice (33), with aging effects on proteins involved in energy metabolism significantly reversed by either treatment. To test whether ACA treatment had similar effects on age-related changes in the cardiac proteome in old UM-HET3 mice, and whether such effects were sex specific, we conducted proteomic analysis on heart tissue from young, old control, and old ACA-treated mice of both sexes. We compared proteins that changed in abundance with age in animals from 6 to 25 months in untreated control animals by 2-way ANOVA, identifying proteins that differed in abundance according to age or sex, or showed an interaction effect between these factors. We detected peptides that mapped to 1389 unique proteins but were unable to detect any proteins that showed a significant difference in abundance between young and old mice after correction for FDR in the 2-way ANOVA or that differed significantly with age when considering males and females separately (Figure 3H and Supplemental Data Set 2). Age-associated changes in cardiac hypertrophy observed in this study are therefore not associated with a change in the cardiac proteome. Furthermore, changes previously reported with age in 29-month-old female C57BL/6J mice are not observable in a consistent manner in 25-month-old UM-HET3 animals (33).

In addition to testing for aging effects on the cardiac proteome, we also tested whether ACA altered the abundance of specific proteins and whether protein changes were associated with specific biological processes or cellular compartments. This analysis involved only samples from 25-month-old animals and compared either untreated animals or those treated with ACA from 4 months of age. When testing for an effect of ACA in a 2-way ANOVA including sex and treatment as factors, we observed 16 proteins that were significantly altered in abundance with ACA treatment after correction for FDR. Thirteen proteins were significantly increased in abundance with ACA treatment, and 3 were decreased in abundance, all of which showed a similar change across sexes (Figure 3G). It was notable that the majority of the proteins increased in abundance were functionally involved in lipid metabolism and proximally related to the peroxisome. To test whether any of these proteins were associated with a particular cellular component, we used an overrepresentation analysis (34). This statistical analysis tests whether groups of proteins linked to a specific compartment or process are more overrepresented than expected by chance within the list of significantly changed proteins, when compared with all-identified proteins, with FDR correction based on the number of group comparisons. This analysis showed that proteins located within the peroxisomes were more represented in the list of proteins increased in abundance with ACA treatment than expected by chance (PANTHER overrepresentation test: *P* < 0.001 after correction for FDR; 10 of 39 proteins detected in the analysis and associated with the peroxisome were significantly increased with ACA, whereas only 0.5 of 39 proteins would be expected to differ with treatment at the *P* < 0.05 criterion as a consequence of type I error; Figure 3H).

### ACA remodels the cardiac lipidome and reduces age-associated lipid changes in a sex-specific manner.

**Figure 4:**
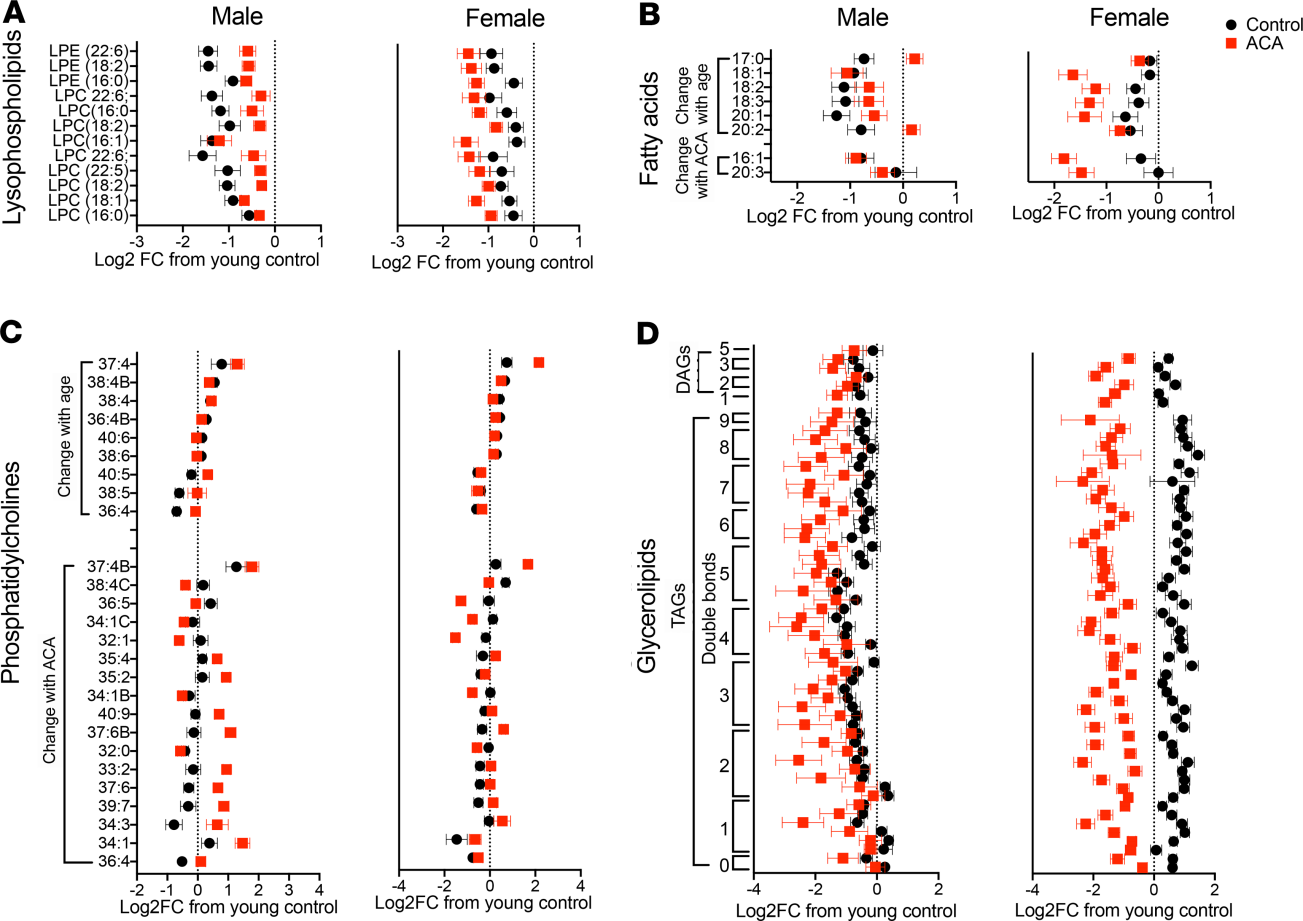
Effect of aging and ACA on the cardiac lipidome. Young animals used for the aging comparison were 4 months of age, and old animals were 22 months (*n* = 8 per sex for each group). Individual lipid species changed significantly with age and/or ACA treatment after correction for FDR. Data for old control mice and old control mice treated with ACA are presented in relation to the log_2_ fold change (Log2FC) when compared with the average of young control animals. Points denote the average Log2FC change, and data are presented as mean ± SEM. (**A**) Lysophospholipids (LPCs) were significantly decreased with aging in both sexes, and this effect was reversed in males treated with ACA but not females. (**B** and **C**) Age-associated changes in fatty acid species and a subset of phosphatidylcholines (PCs) were also reversed in males but not females. (**D**) Glycerolipids (DAGs and TAGs) were significantly reduced with ACA treatment but were not changed with age.

Given the increase in abundance of cardiac proteins involved in lipid metabolism with ACA treatment, we conducted lipidomics analysis of heart tissue from a separate cohort of young mice, 22-month-old mice and 22-month-old mice treated with ACA. This allowed us to test whether ACA has direct effects on the abundance of lipid species and whether ACA ameliorates (or prevents) changes in the cardiac lipidome that occur with age. Using the same 2-way ANOVA approach as with the proteomics data set to identify lipid species that change in abundance with age, we found aging influenced the abundance of 36 lipid species, with age-associated changes that were similar in both sexes (Table 1). ACA opposed the effects of aging of over 50% of these lipids, although the effect differed depending on sex, with ACA having opposing effects on the majority of lipid species in male but not female mice (Table 1, as shown by the significant interaction between ACA × sex). While both sexes showed a decline in lysophosphatidylcholines (LPCs) with aging (Figure 4A), levels of LPCs were decreased further in female mice treated with ACA (an exaggeration of the age effect) but increased in male mice undergoing ACA treatment. LPC abundance values were similar to young controls in ACA-treated male mice (Figure 4A). Notably, LPC 18:2 concentration decreased with age, and this decline was ameliorated by ACA treatment in males. Lyso-PC18:2 has also been shown to decrease with age in humans (35), with low levels associated with poor age-related health, including lowered gait speed (36). Some lysophosphatidylethanolamine (LPEs) were also reduced with aging, and these effects were significantly ameliorated in males treated with ACA but not in female mice (Figure 4A). We also observed a decline in the abundance of several medium chain fatty acids with aging, which increased in male mice and decreased in female mice treated with ACA (Figure 4B). The relative abundance of several phosphatidylcholines (PCs) also changed with age, with some increasing and others decreasing. These age-associated changes were also partially ameliorated by ACA treatment in males (Figure 4C).

We also tested whether ACA influenced the abundance of cardiac lipids at 22 months of age without reference to abundance in young animals, comparing old animals and old animals treated with ACA. This analysis was conducted across all lipid species identified in the heart, not just those that changed significantly with age. The 2-way ANOVA showed that ACA had strong effects on the cardiac lipidome, when considering all detected and identified species, with 80 of 380 lipid species showing a significant change with ACA treatment after correction for FDR (Supplemental Data Set 3). This contrasts with 40 species that differ according to sex in the 2-way ANOVA. The most strongly affected lipid class was triglycerides (TAGs), a lipid class that is implicated in cardiovascular disease (37). TAGs decreased in abundance with ACA treatment (Figure 4D). None of the detected lipid species, including TAGs, showed a significant sex by treatment interaction — although, visually, effects of ACA appear more consistent in females. None of these TAGs were identified as changing significantly with age in the first 2-way ANOVA model (Table 1). Most PCs increased with ACA, although 4 decreased in abundance: PC32:0, PC32:1, PC34:1, and PC36:5C. Saturated and monounsaturated forms of PC32 and PC34 have been positively associated with mortality risk in humans (38); thus, reductions in the abundance of these forms could conceivably have protective effects on cardiac function.

### ACA reduces liver pathology in both sexes.

**Figure 5:**
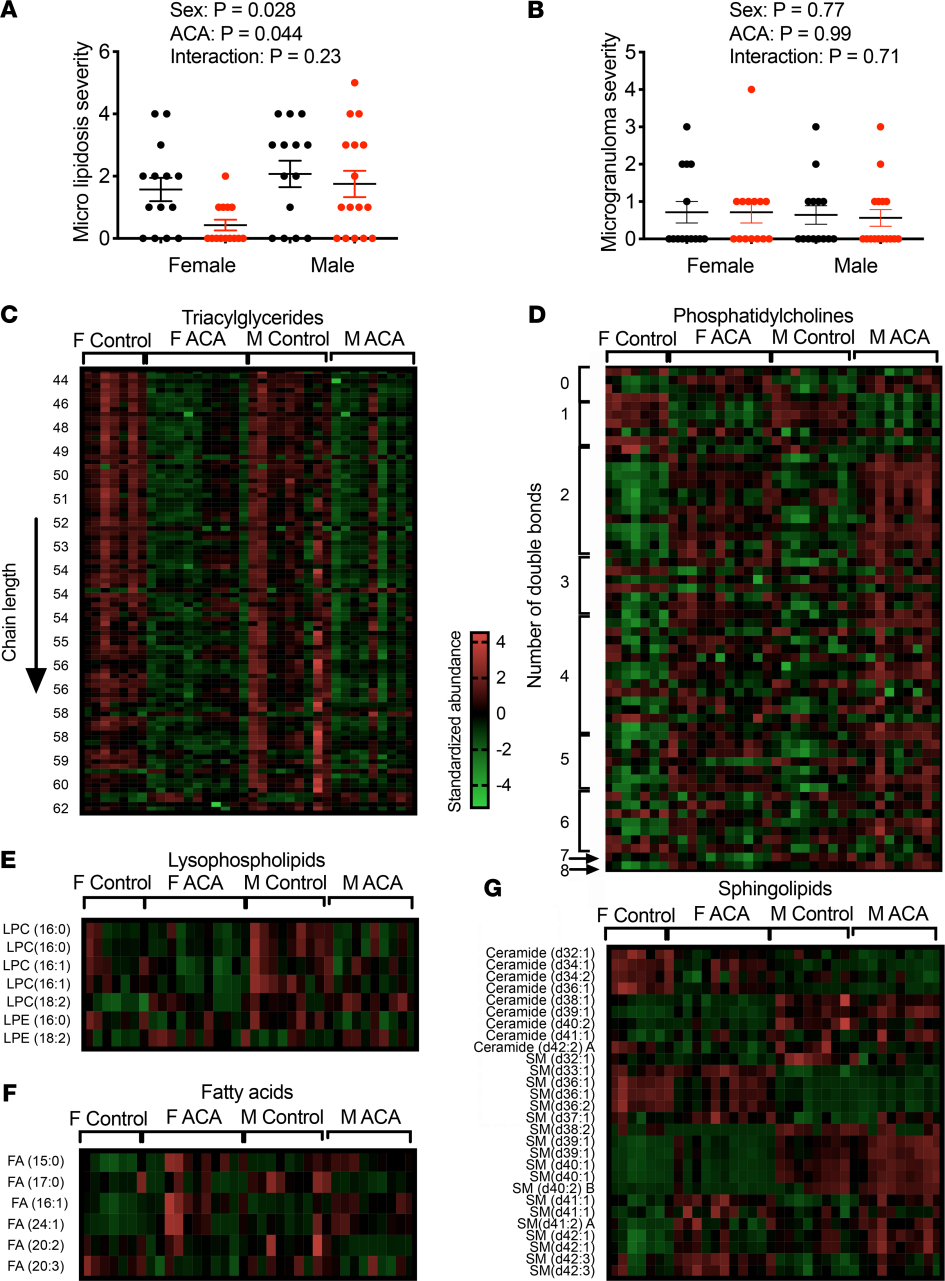
ACA reduces hepatic lipidosis and alters the hepatic lipidome similarly in both sexes. (**A** and **B**) Liver pathology scores are from 25-month-old mice, with each dot representing a data point for an individual animals. (**C**–**G**) Lipidomics was conducted on 12-month-old animals. Sample sizes for lipidomic analysis were 7 female controls, 10 ACA females, 9 male controls, and 9 ACA males. (**C**) Heatmap of TAG species significantly changed in abundance with ACA treatment after correction for FDR. Each row represents a different TAG species, with rows ordered according to the chain length of each species (every fifth row labeled). Values for each species have been standardized before plotting, showing the number of SDs that each observation falls above or below the mean. Each column represents a value for a different mouse. (**D**) Changes in phosphatidylcholines in ACA-treated mice. Each row represents an individual species that differs in abundance with ACA treatment, with rows ordered according to number of double bonds. (**E**–**G**) Lysophosphatidylcholines (LPC), Lysophosphatidylethanolamines (LPE) (**E**), fatty acids (**F**), and sphingolipids (**G**) significantly altered in abundance with ACA treatment. Statistics calculated with a 2-way ANOVA, including sex and treatment as variables.

Previous research suggests that ACA-treated male mice, at time of death, show fewer incidental liver lesions (lesions that did not contribute to a moribund state or the death of animal) compared with control animals, and control male mice have a higher proportion of incidental liver lesions than females (2). We tested whether ACA reduced the incidence of liver pathology when animals are evaluated at a controlled time point (25 months). Liver samples from ACA and control mice were scored by a veterinary pathologist (blind to sex and treatment group) for detectable liver pathologies (lipidosis, necrosis, inflammation, microgranulomas, lymphoid, and Kupffer cell activation) on a scale from 0 (not present) to 5 (very severe), similar to previous scoring for long-lived mice (39, 40). The 2 pathologies most frequently observed were microlipidosis and microgranulomas. Hepatic microlipidosis was scored as more severe in males than females, and ACA significantly reduced the severity as defined by this score, similarly in both sexes (Figure 5A). Evidence of microgranulomas was observed in approximately 50% of animals at 25 months, but the severity of this was not influenced by sex or ACA treatment (Figure 5B). All other pathologies were detected in less than 3 individuals per treatment and, therefore, were not analyzed for ACA effects.

ACA can cause changes in the liver metabolome of mice, although little is known about the specific lipid metabolites that change in response to treatment and correlate with reduced liver pathology (41). We tested whether ACA altered the abundance of a variety of lipid species in mice and whether these effects were dependent on sex. This analysis was conducted on animals at 12 months of age, so we could not evaluate possible aging effects. ACA caused a remarkable restructuring of the lipidome of mice at 12 months of age, with 237 of 442 identified lipid species significantly changing in abundance with ACA (*P* < 0.05 after correction for FDR). This contrasts with 80 species that differ significantly according to sex. None of these metabolites showed a sex by treatment interaction after correction for FDR, indicating that responses in liver lipids occur similarly in both sexes.

When assessing changes in hepatic lipid classes, the most notable change was the major reduction in most TAGs. Eighty-eight species significantly decreased in abundance with ACA, and 5 increased (Figure 5C). The 5 TAGs increasing in abundance were long-chain TAGs 54:5, 58:10, 60:12, 62:13, and 62:14 (Figure 5C). We also observed a major restructuring of the composition of PCs, with a reduction in the abundance of monounsaturated PCs and an increase in most polyunsaturated PC species (Figure 5D). This shift is consistent with the changes observed in the heart and the association between monounsaturated PCs and hepatic steatosis (42). Several lysophospholipids also change in abundance with ACA. LPC 18:2 and LPE 18:2 increased in abundance, as in the heart, while LPCs with a chain length of 16 decreased (Figure 5E). Fatty acids and sphingolipids were the other 2 lipid groups notably affected by ACA in the liver. ACA causes a change in the relative abundance of many of these species, with some increased in abundance and others decreased (Figure 5, F and G). A full list of species changing with ACA treatment is shown in Supplemental Data Set 4.

## Discussion

ACA increases life span in UM-HET3 mice of both sexes, although life span extension is smaller in females (3%–5% change in median life span) than in males (11%–17% change in medium life span, depending on the dose) (2). Our results show that ACA has both sex-dependent and -independent effects on health parameters associated with aging, with physical function improved similarly in both sexes, but several important parameters associated with cardiac health improved with ACA preferentially in males.

One of the sex-independent effects of ACA was a reduction in body weight recorded over a middle-age time point up to 12 months. The early-life weight reduction observed is presumably attributed to loss of fat mass, since ACA has been reported to reduce fat mass in mice (22). This previous study reported site- and sex-specific changes in body composition, although comparison of relative changes in different sexes was qualitative and no formal tests of differing responses to ACA between sexes were made (22). We sampled mice monthly from 4 to 25 months of age, allowing us to test whether the effects ACA on body weight changed with age and differed between sexes. At later ages (12–18 and 18–24 months), ACA protected against age-associated weight loss in both male and female mice. The underlying mechanism of this protection seemed to differ between sexes, in that dissection of animals at 25 months revealed preservation of quadriceps mass in male mice treated with ACA but not in females. Preservation of muscle mass during aging is particularly important for protecting against disability and frailty (43); the use of ACA could have further benefits in aging T2DM patients, who may have accelerated loss of muscle mass and strength as a result of their disease (44).

We also evaluated ACA effects on a series of clinically relevant measures that assess balance/coordination capacity, endurance, and strength (45). Justice et al. (45) show that these measures all declined with age in C57BL/6J male mice. We found a decline with age for fall latency on an accelerating rotarod and rotarod endurance, as well as grip strength, test in mice of both sexes. Treatment with ACA ameliorated the age-related decline in all these measures independently of sex, in contrast to the sexual dimorphism in the ACA effect on life span, which is stronger in males (2). In the accelerating rotarod test, we identified body weight as a covariate that accounted for the effects of ACA on fall latency. In contrast, ACA improvements in rotarod endurance capacity and on grip strength remained significant, even when adjusting for body weight. This suggests that ACA confers benefits that contribute to healthy aging that are not explained solely by changes in weight.

In the heart, we observed age-related pathologic changes in male mice, including well-defined structural remodeling that was lessened by ACA treatment. Left ventricular hypertrophy and diastolic dysfunction occur with age in humans independently of sex (46) and contribute to cardiac-specific morbidities, including cardiovascular disease and heart failure (47). Mouse models can recapitulate cardiac aging phenotypes (48), although the effects of sex on these changes has not been well described. Hypertrophy of the left ventricle and increased posterior wall thickness were present in aged male UM-HET3 mice, and this effect was diminished in ACA-treated males. We also noted prolongation of IVRT with age in male mice, but this change was not significantly affected by ACA treatment. Our evaluation may have been too early to detect age-dependent diastolic dysfunction, in that other reports conducted assessment at time points after 25 months of age (33, 49). The lack of change in female hearts with ACA treatment may also be because females showed less overall dysfunction than males at 25 months of age, and it’s possible that responsiveness to ACA would be more apparent at later ages. However, at ages later than 25 months, selective disappearance can be a problem, with some control animals dying before sampling when compared with longer-lived drug-treated animals. The time point selected allowed us to determine whether there were cardiac structural or functional changes that precede this dysfunction and whether this is influenced by ACA treatment.

Previous studies have identified declines in cardiac proteins involved in lipid and fatty acid metabolism with age in mice (33, 49), which could be linked to the shift away from fat oxidization and linked to impaired cellular and organ function (50). Age-related declines in proteins involved in fatty acid metabolism are ameliorated by short-term rapamycin or caloric restriction treatment (33). In our study, we did not observe consistent age-associated changes in cardiac protein abundance. We used a similar proteomics approach to previous work in female C57BL/6J mice and had a similar sample size, although our cohorts of young and old mice were spread equally across 2 sexes. The lack of a strong age-associated signature in proteome (sufficient to be detectable after correction for FDR) could be because we sampled animals 2–3 months earlier in life or because we studied a different, genetically heterogeneous strain of mouse. The old animals used on this comparison also underwent a brief sham surgery at 3 months of age, including anesthesia with tribromoethanol. However, it seems unlikely that a single sham surgery 2 years prior would obscure any age-associated change in the cardiac proteome, and the effects of ACA in this cohort fit with those seen in the other ACA cohorts. ACA did, however, increase the abundance of several proteins in the heart, and overrepresentation analysis showed significant enrichment in peroxisomal proteins among those increased in abundance by ACA (34). From static measures of protein abundance, it is not possible to ascertain the functional significance of these changes for lipid metabolism or other peroxisomal processes. To test whether these responses might be linked to age-associated changes in lipid abundance, we examined changes in the cardiac lipidome with age and whether these responses were ameliorated with ACA.

While we failed to detect a change in the cardiac proteome with age, our lipidomic analysis detected an age-associated shift in the abundance of several lipid classes in the left ventricle, highlighting that lipidomic responses to aging in the heart are detectable even before the occurrence of any clear cardiac dysfunction. Most notably, the abundance of cardiac LPCs was reduced with age in both sexes, and lifelong ACA treatment lessened this reduction — specifically in male mice. Recent findings have shown that male patients with atherosclerotic disease have low serum levels of LPCs, including LPC 18:2 (51), which also decline with age and are associated with compromised physical function (35, 36). ACA has been shown to reduce risk of cardiovascular disease and mortality in certain populations (e.g., patients with T2DM) (52, 53), and the alteration of LPCs may represent a potential mechanism by which ACA can maintain or improve cardiac and cardiovascular health over time. We also note that female mice tended to show the opposite change in the abundance of specific LPC species with ACA, and for some, the effects of aging appear to be exacerbated after treatment; however, cardiac function was not detectably altered. This suggests that there is not a simple linear relationship between cardiac LPC abundance and cardiac function that is observable across both sexes in mice over the time course examined. It is possible that effects of ACA on cardiac function, either beneficial or deleterious, may be detectable at later ages in females and/or that LPCs are related to cardiac function in a sex-specific way, with a possible greater importance in males.

We observed that ACA treatment decreased cardiac TAGs in both male and female mice. Accumulation of TAGs in the heart occurs with atherosclerosis and related risk factors (i.e., obesity and T2DM), which can induce cardiac lipotoxicity and impair cardiomyocyte structure and function (54). The slowing of age-associated changes in PCs and fatty acids may also provide benefits to cardiac health in aging males, particularly given the role of fatty acids as the primary cardiac metabolic fuel source and the structural alterations in PCs that occur with pathological cardiac hypertrophy (55). Taken together, our data suggest that ACA may play a beneficial role in preserving lipid utilization and cardiac structure, leading to improved cardiac health and function over time.

We observed significant alterations of the liver lipidome in both sexes as a result of ACA treatment at middle age (12 months) and a parallel reduction in hepatic lipidosis as detected through histopathological measures at a later age (25 months). ACA reduces serum TAGs (56) and low-density lipoprotein cholesterol (LDL-C) (57) in T2DM patients, and our data indicate that it also has a major restructuring effect on lipid species in the liver of nondiabetic mice. The reduction in TAGs may be particularly relevant in the context of cardiovascular disease, given that accumulation of fat in liver can lead to nonalcoholic fatty liver disease (NAFLD), which is an independent risk factor for cardiovascular disease (58).

Overall, our data suggest that ACA has a significant influence on the lipid milieu of the heart and liver. We have previously shown that ACA has a sex-specific effect on mTORC2 signaling in the liver of 12-month-old animals, such that males show an increase in mTORC2 signaling with ACA but females show no change (59). Given the central role of mTORC2 in regulation of lipid metabolism (60), it could be assumed that changes in this signaling pathway may help shape the alterations in lipid profile that occur with ACA. Alternatively, mTORC2 signaling can itself be altered by lipids (60), so changes in the accumulation and/or composition of lipids (for example, those in the liver accumulating from circulation) may influence mTORC2 activity. It is important to recognize, however, that the lipidomic changes in the liver occur similarly in both sexes, while increased mTORC2 signaling occurs specifically in males, correlating with male-specific improvements in glucose tolerance and life span. Thus, activation of mTORC2 may not be causally related to the altered lipid profile, or sex-specific factors like sex-hormones may influence the relationship between mTORC2 activation and lipid biology, obscuring any relationship from being observed across both sexes.

In the heart at 25 months, we also observed that phosphorylation of AKT and SGK1 at sites regulated by mTORC2 is reduced, consistent with lower mTORC2 signaling in males — the opposite pattern of mTORC2 activation in the liver at 12 months. To confidently determine whether changes in mTORC2 activity do occur with ACA in the heart would require a comprehensive analysis of both mTORC2-sensitive and -nonsensitive substrates, which was not undertaken in this study. However, the activation of both mTOR complexes varies across life in tissue-specific ways (61); therefore, both increases and decreases in mTOR signaling may help prevent dysfunction with aging. Cardiac mTORC2 signaling, while important for cardiomyocyte growth in young animals (62), may be detrimental for cardiac function in old animals, particularly given the role of AKT activation in promotion of pathological cardiac hypertrophy (29). It is possible that suppression of mTORC2 signaling in old animals may be beneficial for cardiac function and could contribute to the benefits of late-life rapamycin treatment (33), which suppresses both mTORC1 and mTORC2 signaling when applied chronically over a sustained treatment period. Ultimately, tissue-specific manipulation of the activity of both complexes is required to determine their causal role in lipid metabolism and age-associated pathology in individual tissues.

Our data highlight the potential of ACA to improve a variety of aspects of health in aging mice and make this treatment worthy of consideration as an anti-aging treatment that could be used to improve human health in both diseased (e.g., diabetic and prediabetic) and nondiseased individuals. The longevity and pathology data, previously published (2), show that most UM-HET3 mice die of some form of neoplastic disease and that these cancer-dependent deaths are delayed in ACA-treated mice, particularly in males. The current paper shows that similar benefits accrue in aging mice for physical function, cardiac, and metabolic indices, strongly supporting the hypothesis that ACA may be able to alter multiple aspects of aging, independently of its role as an antidiabetic therapy.

## Methods

### Animals.

UM-HET3 mice were produced as previously described (5, 63). The mothers of the test mice were CByB6F1/J, JAX stock no. 100009, whose female parents are BALB/cByJ and whose male parents are C57BL/6J. The fathers of the test mice were C3D2F1/J, JAX stock no. 100004, whose mothers are C3H/HeJ and whose fathers are DBA/2J. Mice in breeding cages received Purina 5008 mouse chow, and weaned animals were fed Purina 5LG6. Mice were housed as previously described (5, 63, 64) in plastic cages with metal tops, using quarter-inch corn-cob bedding (Bed O’Cobs, Andersons Lab Bedding). Mice were given free access to water via bottles. Mice were housed in ventilated cages and were transferred to fresh cages every 14 days. Temperature was maintained within the range of 21°C–23°C.

### Diets.

At 4 months of age, animals in different sibling groups were randomly allocated to control or ACA treatment. Animals in the control group remained on the 5LG6 diet, while ACA animals were switched to a diet containing ACA added to 5LG6. Diets were prepared by TestDiet Inc. (Purina Mills). ACA was purchased from Spectrum Chemical Mfg. Corp. and was mixed at a concentration of 1000 mg of ACA per kilogram of diet (1000 ppm) — a dose that has been shown to achieve life span extension by the NIA Interventions Testing Program (2).

### Cohorts.

Three separate cohorts of animals were used to gather the data, and all cohorts were treated with ACA using the same protocol and starting at the same age. One cohort was used for physical function, echocardiography, and cardiac histology measures, which were conducted when mice were 22 months old. A second cohort was used for cardiac lipidomics and body temperature measures, and the mice in this cohort were sampled at 22 months of age. A third cohort was sampled for monthly body weight, and a subset was euthanized for liver lipidomics at 12 months of age, after which the surviving animals were euthanized at 25 months of age for assessment of organ weights, cardiac signaling and proteomics, and liver pathology. This third cohort of mice also went through a brief sham surgery at 3 months, where males were sham castrated (incision made in scrotal sac; testicles exteriorized and then replaced) and females were sham ovariectomized (incision made on the left side perpendicular; ovary and fat pad were exteriorized and replaced). For this sham surgery, the mice were anesthetized by injection tribromoethanol (250 mg/kg; MilliporeSigma) and given a single preoperative injection of the analgesia carprofen (5 mg/kg; Rimadyl). Full details are given in Garratt et al. (59). Where relevant, at the sampling point of each cohort, we also included a comparable group of unoperated young controls aged between 4 and 6 months old. These animals were bred using the same scheme as old animals and were maintained on the 5LG6 diet from weaning until sampling.

### Animal dissections.

Animals were dissected in the morning (9:00 a.m.–noon) after 18 hours of fasting. Tissues were weighed, frozen in liquid nitrogen, and stored at –70°C unless otherwise stated. For assessment of cardiac signaling proteins and proteomics, the whole heart was cleaned of excess blood with PBS and then frozen. For lipidomics, the left ventricle was dissected and used for analysis. For liver pathology, approximately one-third of the liver was immediately stored in 10% buffered formalin.

### Liver pathology analysis.

Liver pathology was assessed using standard microhistopathological methods by a board-certified veterinary pathologist who was blinded to sex and treatment group when conducting the assessment. This pathologist recorded all detectable liver pathologies (lipidosis, necrosis, inflammation, microgranulomas, lymphoid, and Kupffer cell activation) and scored them on a scale from 0 (not present) to 5 (severe), similar to previous scoring for long-lived mice (39, 40). Pathology analysis was conducted on all mice that were euthanized for planned dissections at 25 months of age and included 14 female control mice, 14 ACA female mice, 14 male control mice, and 16 ACA male mice.

### Cardiac fibrosis.

Following euthanasia, mouse hearts were perfused with PBS until lungs and liver were clear. Hearts were dissected, and 2 mm of the apex was placed in 10% neutral buffered formalin solution (MilliporeSigma) for 24 hours; it was then transferred to 70% ethanol. The heart apex was sent to the Histology Core at UM School of Dentistry. Heart apices were paraffin embedded, sectioned, and stained with Picrosirius red. Following histological processing, imaging was conducted at UM Microscopy Core on an Olympus BX51 Upright Light Microscope. For each mouse, left and right sides of 3 of 10 random slices were selected for imaging at 20× magnification. Fibrosis was measured as a percentage of the area of collagen deposition using ImageJ software (NIH). Samples numbers include *n* = 8 young male controls, *n* = 8 old (22-month) male controls, and *n* = 12 old ACA (22-month) males.

### Cardiac growth–promoting pathways.

Activation of growth-promoting pathways in the heart was assessed by Western blot, using antibodies for total protein and phospho-specific antibodies, with 6 samples included per sex, per treatment group. Tissues were processed, whole-cell lysates were obtained, and equal amounts of protein were loaded for Western blot analysis. Antibodies and phospho-specific rabbit antibodies were purchased from Cell Signaling (pAKT 473, 4060; total AKT, 9272; www.cellsignal.com), Santa Cruz Biotechnology Inc. (pSGK1, 16745; www.scbt.com), and Genetex (SGK1, 61249; www.genetex.com). Protein ubiquitination was also assessed via Western blot using an antibody purchased from Cell Signaling Technology (catalog 3933).

### Physical function testing.

Physical function testing was conducted at the UM Physiology Phenotyping core within a single, 1-week period and with a rest day between each test for each mouse. Testing was randomized, blinded, and adapted from Justice et al. (45). Grip strength was assessed using a Grip Strength Meter (Columbus Instruments). Average kilograms of force for 5 forelimb grip strength trials per mouse were recorded with 5 minutes of rest between trials.

Rotarod testing was performed using a Dual Species Economex Rotarod (Columbus Instruments) following previously published protocols for assessment of age-associated changes in physical function (45). For balance/coordination, initial speed was 5 rpm and rotarod was accelerated at 0.1 rpm/s. Fall latency was recorded by the Rotarod timer over 3 trials with 1-minute rest between trials. Average fall latency over the 3 trials was used for analysis. For endurance testing, the rotarod speed began at 5 rpm and was accelerated at 0.1 rpm/s over 15 minutes until the speed reached the group-specific average speed achieved during acceleration testing (young average, 24 rpm; old average, 16 rpm). From this point, mice could continue for an additional 15 minutes for a total potential run time of 30 minutes. Mice were allowed up to 10 falls within the first 15 minutes and up to 2 falls in the remaining 15 minutes. Fall latency was scored once the mouse fell more than the permitted criteria. Samples numbers include *n* = 8 young female controls, *n* = 8 old (22-month) female controls, *n* = 12 old ACA (22-month) females, *n* = 8 young male controls, *n* = 8 old (22-month) male controls, and *n* = 12 old ACA (22-month) males.

### Core body temperature.

Core body temperature was assessed using a thermistor rectal probe (Braintree Scientific). Mice were restrained in supine position. Rectal probe was lubricated with glycerol and inserted ~1–2 cm into rectum for approximately 10 seconds. Temperature was recorded to the nearest 0.1°C. The probe was cleaned with ethanol wipes between mice. Sample numbers include *n* = 16 young female controls, *n* = 34 old (22-month) female controls, *n* = 18 old ACA (22-month) females, *n* = 12 young male controls, *n* = 16 old (22-month) male controls, and *n* = 16 old ACA (22-month) males.

### Echocardiography.

Echocardiography was performed at the UM Physiology Phenotyping Core by a single trained technician. All testing was randomized and blinded. Induction of anesthesia was performed in an enclosed container filled with 5% isoflurane. After induction, the mice were placed on a warming pad to maintain body temperature. A total of 1%–1.5% isoflurane was supplied via a nose cone to maintain a surgical plane of anesthesia. The hair was removed from the upper abdominal and thoracic area with depilatory cream. ECG was monitored via noninvasive resting ECG electrodes. Transthoracic echocardiography was performed in the supine or left lateral position. Two-dimensional, M-mode, Doppler, and tissue Doppler echocardiographic images were recorded using a Visual Sonics’ Vevo 2100 high-resolution in vivo microimaging system. Sample numbers include *n* = 8 young female controls, *n* = 8 old (22-month) female controls, *n* = 12 old (22-month) females treated with ACA, *n* = 8 young male controls, *n* = 8 old (22-month) male controls, and *n* = 12 old (22-month) males treated with ACA.

### Cardiac proteomics.

Proteomics analysis was conducted with liquid chromatography–tandem mass spectrometry (LC-MS/MS) analysis using a Thermo Easy nano-LC and an Orbitrap Fusion Mass Spectrometer. Each sample batch consisted of 24 individual mouse heart tissue samples, representing 4 randomly selected mice from 6 groups and 2 pooled references, for a total of 26 samples per batch. The first reference was a pool composed of 32 samples — 4 randomly selected mice from 8 groups (2 of the 8 groups not represented in this study). The second reference was a pool composed of 24 samples — 4 randomly selected mice from 6 groups (none of the 6 groups represented in this study). See Supplemental Methods for full methods.

### Lipidomics.

Lipidomic data were acquired at the NIH West Coast Metabolomics Center at the UCD, using an untargeted analysis for complex lipids via charged surface hybrid column–quadrupole time of flight mass spectrometer (CSH-QTOF MS/MS). For lipid analysis of heart tissue, we included 8 samples per sex per treatment. For liver tissue, we conducted analysis on 7 female control mice, 10 ACA female mice, 9 male control mice, and 9 ACA male mice, representing all samples available from this 12-month time point.

### Statistics.

Unless otherwise noted, our statistical approach was to conduct a 2-way ANOVA. For analysis of traits that changed with age, we examined responses in untreated animals, including factors for age (young or old) and another for sex (male or female), as well as their interaction. For analysis of traits in relation to ACA treatment, we restricted analysis to old animals and included a treatment factor (ACA or control), another for sex (male or female), and an interaction between the two. If there was a significant interaction between ACA and sex, we followed up by conducting a 2-tailed Student’s *t* test to determine whether there was a significant effect of ACA within each sex. In situations where old control animals of one sex where separately compared with young animals and old ACA-treated animals (because one of the above mentioned interactions was significant), we conducted a Dunnett’s multiple comparison test to calculate *P* values for these 2 comparisons.

For proteomic and lipidomic data sets, because multiple comparisons were conducted across large data sets, we adjusted raw *P* values with the Benjamini-Hochberg FDR method (65), as previously conducted in the context of other drug treatments with sex-specific life span effects (66). Overrepresentation analysis was conducted using the PANTHER overrepresentation test, using the Gene Ontology (GO) Cellular Component Complete Annotation Set (34).

A 2-way ANOVA of proteomic and lipidomic data sets was conducted in MetaboAnalyst (67). All other analyses were conducted in SPSS version 24, using the general linear model function and a full factorial model. In models where we were interested in controlling for effects of body weight, we included body weight as a continuous covariate (e.g., an ANCOVA was conducted). To understand whether ACA influences body weight changes over different periods of life, we examined the change in body weight between specific time points. We included all available data from individuals that were alive at each time point, with *n* = 17 female controls at 12 and 18 months, *n* = 14 at 24 months; *n* = 19 ACA female at 12 and 18 months, *n* = 16 at 24 months; *n* = 16 for control males at 12 and 18 months, *n* = 14 at 24 months; *n* = 18 ACA male at 12 and 18 months, and *n* = 16 at 24 months.

Data were visually inspected to ensure conformation to parametric assumptions. Proteomic and lipidomic data sets were transformed logarithmically before analysis. Liver pathology scores were ranked, and rank scores were used for analysis.

## Author contributions

JJH, MJM, SMD, RAM, and MG designed research; JJH, SL, KP, DL, GEM, JHP, KS, JW, JEW, OF, and MG performed research; JJH and MG analyzed data; and JJH and MG wrote the paper.

## Supplementary Material

Supplemental data

Supplemental Data Set 1

Supplemental Data Set 2

Supplemental Data Set 3

Supplemental Data Set 4

## Figures and Tables

**Table 1:**
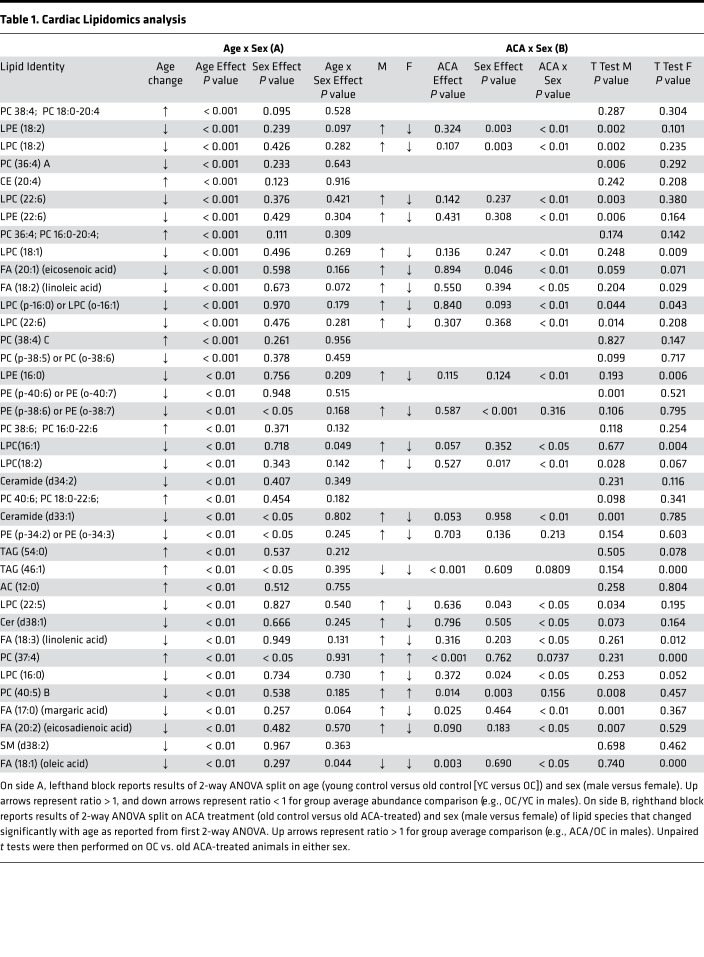
Cardiac Lipidomics analysis

## Version 1 09/29/2020

In-Press Preview

## Version 2 11/05/2020

Electronic publication
