# Supplementary material for: Acarbose has sex-dependent and -independent effects on age-related physical function, cardiac health, and lipid biology
Source: JCI Insight. 2020 Nov 5;5(21):e137474. doi: 10.1172/jci.insight.137474 (PMC7710286; doi:10.1172/jci.insight.137474)
Supplement: Supplemental data [file jciinsight-5-137474-s214.pdf]

Supplementary Methods

Proteomic and Lipidomic analysis

Cardiac proteomics

Batch Design and References

Each sample batch was 24 individual mouse heart tissue samples, representing 4 randomly selected mice from 6 groups, and 2 pooled references for a total of 26 samples per batch. The first reference was a pool composed of 32 samples, 4 randomly selected mice from 8 groups (2 of the 8 groups not represented in this study). The second reference was a pool composed of 24 samples, 4 randomly selected mice from 6 groups (none of the 6 groups represented in this study). Both references were homogenized, aliquoted, frozen, and used to compare between batches and as a "universal reference" for comparison between similar proteomic studies.

Lysis/Digestion

Mouse heart tissue previously probe sonicated in 90 ul of 0.1% RapiGest in 100 mM Tris with phosphatase inhibitors was resuspended in 30 ul of 3X lysis buffer for a final concentration of 5% SDS, 50mM Triethylammonium bicarbonate (TEAB), 2mM $MgCl_2$, 1X HALT phosphatase and protease inhibitors and vortexed. Protein concentration was measured with a BCA assay. Homogenate of 50 ug was added to a process control of 800 ng of yeast enolase protein (Sigma) which was then reduced with 20 mM DTT and alklyated with 40 mM IAA. Lysates were then prepared for S-trap column (Protifi) binding by the addition of 1.2% phosphoric acid and 350 ul of binding buffer (90% Methanol, 100 mM TEAB). The acidified lysate was bound to column incrementally, followed by 3 wash steps with binding buffer to remove SDS and 3 wash steps with 50:50 methanol:chloroform to remove lipids and

a final wash step with binding buffer. Trypsin (1:10) in 50mM TEAB was then added to the S-trap column for digestion at 47°C for one hour. Hydrophilic peptides were then eluted with 50 mM TEAB and hydrophobic peptides were eluted with a solution of 50% acetonitrile in 0.2% formic acid. Elutions were pooled, speed vacuumed and resuspended in 0.1% formic acid. A heavy labeled Peptide Retention Time Calibrant (PRTC) mixture (Pierce) was added to each sample.

Liquid Chromatography and Mass Spectrometry

One ug of each sample with 150 femtomole of PRTC was loaded onto a 30 cm fused silica picofrit (New Objective) 75 µm column and 3.5 cm 150 µm fused silica Kasil1 (PQ Corporation) frit trap loaded with 3 µm Reprosil-Pur C18 (Dr. Maisch) reverse-phase resin analyzed with a Thermo Easy nano-LC 1200. The PRTC mixture is used to assess quality of the column before and during analysis. Four of these quality control runs are analyzed prior to any sample analysis and then after every six sample runs another quality control run is analyzed. Buffer A was 0.1% formic acid in water and buffer B was 0.1% formic acid in 80% acetonitrile. The 40-minute QC gradient consists of a 0 to 16% B in 5 minutes, 16 to 35% B in 20 minutes, 35 to 75% B in 1 minute, 75 to 100% B in 5 minutes, followed by a wash of 9 minutes and a 30 minute column equilibration. The 110-minute sample LC gradient consists of a 2 to 7% for 1 minutes, 7 to 14% B in 35 minutes, 14 to 40% B in 55 minutes, 40 to 60% B in 5 minutes, 60 to 98% B in 5 minutes, followed by a 9 minute wash and a 30 minute column equilibration. Peptides were eluted from the column with a 50°C heated source (CorSolutions) and electrosprayed into a Thermo Orbitrap Fusion Lumos Mass Spectrometer with the application of a distal 3 kV spray voltage. For the quality control analysis, a cycle of one 120,000 resolution full-scan mass spectrum (350-2000 m/z) followed by a data-independent MS/MS spectra on the loop count of 76 data-independent MS/MS spectra using an inclusion list at 15,000 resolution, AGC target of 4e5, 20 sec maximum injection

time, 33% normalized collision energy with a 8 m/z isolation window. For the sample digest, first a chromatogram library of 6 independent injections is analyzed from a pool of all samples within a batch. For each injection a cycle of one 120,000 resolution full-scan mass spectrum with a mass range of 100 m/z (400-500 m/z, 500-600 m/z…900-1000 m/z) followed by a data-independent MS/MS spectra on the loop count of 26 at 30,000 resolution, AGC target of 4e5, 60 sec maximum injection time, 33% normalized collision energy with a 4 m/z overlapping isolation window. The chromatogram library data is used to quantify proteins from individual sample runs. These individual runs consist of a cycle of one 120,000 resolution full-scan mass spectrum with a mass range of 350-2000 m/z, AGC target of 4e5, 100 ms maximum injection time followed by a data-independent MS/MS spectra on the loop count of 76 at 15,000 resolution, AGC target of 4e5, 20 sec maximum injection time, 33% normalized collision energy with an overlapping 8 m/z isolation window. Application of the mass spectrometer and LC solvent gradients are controlled by the ThermoFisher XCalibur data system.

Proteomics Data Analysis

Thermo RAW files were converted to mzML format using Proteowizard (version 3.0.19045) using vendor peak picking and demultiplexing. Chromatogram spectral libraries were created using default settings (10 ppm tolerances, trypsin digestion, HCD b- and y-ions) of Walnut in EncyclopeDIA (version 0.9.0) using the Uniprot mouse canonical FASTA. Quantitative spectral libraries were created by mapping spectral to the chromatogram spectral library using EncyclopeDIA requiring a minimum of 3 quantitative ions and filtering peptides at a 1% FDR using Percolator 3.01. The quantitative spectral library is imported into Skyline with the mouse uniprot FASTA as the background proteome to map peptides to proteins.

Data quality control

Mass spectrometry based proteomic data inherently exhibit a right-skewed distribution. Due to the downstream parametric significance tests requiring approximately normally distributed data, log2 transformation is applied and normality is assessed using qualitative graphical measures. Since statistical tests are generally sensitive to the presence of outliers, '-Inf' values and extreme outliers on the lower end of the distribution are identified by one-sided Grubb's test (i.e. maximum normed residual test), rejecting the null hypothesis of no presence of outliers in the data at 0.05 significance level. Outliers identified are treated as missing data for downstream imputation. While deletion of the outlying data points is commonly practiced for erroneous data, the source of the outliers is believed to be fragment ion signals below the detection limit of the mass spectrometer.

Missing value imputation

Missing values are assumed to be the result of the presence of ions below the detectable range of the instrument. Per feature, up to 20% of these missing not at random (MNAR) data are imputed with the left-censored k-nearest neighbor (kNN) averaging method implemented as part of the 'imputeLCMD' R package available through CRAN repository. The optimal value of k is approximated by the square root of the number of samples round to the nearest whole odd number. For this data set, k = 7 is selected as determined by 48 samples. Features with more than 20% missing data are removed from further downstream analysis.

Across sample normalization

Median normalization is applied to the imputed data to account for between-sample variation. This local normalization method increases the precision to which comparison of peptide quantitation can be made across the samples.

Controlling for batch effect and unknown confounders

Surrogate variable (SV) analysis is performed to capture the heterogeneity across the data set caused by batch and unknown confounding effects. Using the ‘sva’ R package available through the Bioconductor framework, 8 SVs are estimated. All of the SVs are included as covariates in the downstream linear model implemented by the ‘limma’ R package to control for the effects of known and unknown sources of unwanted variation.

Protein Quantitation

To avoid assigning the peptide-level quantitation to incorrect protein isoforms, protein quantitation is inferred through parsimoniously assembled peptides aggregated to gene-level using the CPTAC Common Data Analysis Platform (CDAP) protein identification and quantitation methods [64]. Peptide identifications are matched to the associated protein sequences, which in turn are assigned to the respective UniProt and NCBI Gene annotations. The parsimony analysis requires at least two unshared peptides per gene. The label-free gene-level quantitation is inferred by summing peptide-level peak areas. MAYU estimated gene-level FDR was not assessed during the assembly for this particular dataset but is bound by the 1% peptide-level FDR assessed by decoy searches.

DIA Data Availability

The Skyline documents and raw files for DIA library generation and DIA sample analysis are available at Panorama Public. ProteomeXchange ID: PXD017706. Access URL: https://panoramaweb.org/Garratt-ACA.url

Lipidomics

Lipidomic data were acquired at the NIH West Coast Metabolomics Center at the University of California, Davis, using an untargeted analysis for complex lipids via CSH-QTOF MS/MS. For lipid analysis of heart tissue we included eight samples per sex per treatment.

For liver tissue we conducted analysis on 7 female controls, 10 ACA female, 9 male controls and 9 ACA male, representing all samples available from this 12 month time point.

Lipid extraction: Lipids were extracted from 25 µg of plasma. 225 µL cold methanol containing a mixture of 15 deuterated or odd-chain lipid internal standards was added and samples were vortexed for 10 s. After adding 750 µL of MTBE, samples were vortexed for 10 s and shaken for 5 min at 4°C. Next, 188 µL water was added and samples were vortexed for 20 s and centrifuged for 2 min at 14,000x g. One 350 µL aliquot from the non-polar layer was evaporated to dryness in a SpeedVac concentrator. Dried extracts were resuspended using a mixture of methanol/toluene (9:1, v/v) (60 µL) containing an internal standard [12-[[(cyclohexylamino)carbonyl]amino]-dodecanoic acid (CUDA)] used as a quality control. Method blanks and pooled controls were prepared along with the study samples for monitoring the data quality.

LC-MS data acquisition: Samples were analyzed using an Agilent 1290 Infinity UHPLC/6530 QTOF MS or an Agilent 1290 Infinity UHPLC/6550 QTOF MS. A charged surface hybrid (CSH) column C18 2.1×100 mm, 1.7 µm column with a VanGuard CSH pre-column C18 2.1×5 mm, 1.7 µm (Waters, Milford, MA) were used to separate the extracted lipids. A reference solution of purine and HP-0921 (m/z 121.0509, m/z 922.0098 in electrospray ionization (ESI) (+) and m/z 119.0360 and m/z 980.0164 (acetate adducts) in ESI(–)) was used to correct small mass drifts during the acquisition. Mobile phase A (60:40 ACN:water + 10 mM ammonium formate + 0.1% formic acid) was prepared by mixing 600 mL ACN, 400 mL water, 1 mL formic acid and 630 mg of ammonium formate. Mobile phase B solvent (90:10 IPA:ACN + 10 mM ammonium formate + 0.1% formic acid) was prepared by mixing 900 mL IPA, 100 mL acetonitrile, 1 mL formic acid, 630 mg ammonium formate previously dissolved in 1 mL of H_2O. Both solvents were mixed and sonicated for 10 min (twice) before their use. For ESI (–) the composition of mobile phases

was identical but 10 mM ammonium acetate (771 mg per 1 L) was used instead as modifier for the ADNI and GOLDN cohort samples. The quadrupole/time-of-flight (QTOF) mass spectrometers were operated with electrospray ionization (ESI) performing full scan in the mass range m/z 100–1700 in positive and negative modes. Instrument parameters were as follows for the ESI (+) mode on the Agilent 6530 QTOF – gas temperature 325 °C, gas flow 8L/min, nebulizer 35 psig, sheath gas temperature 350 °C, sheath gas flow 11, capillary voltage 3500 V, nozzle voltage 1000 V, and fragmentor voltage 120 V. In negative ion mode (Agilent 6550 QTOF), gas temperature 200°C, gas flow 14L/min, fragmentor 175 V, with the other parameters identical to positive ion mode. Data were collected in centroid mode at a rate of 2 scans/s. Injection volume was 1.7 µL for the positive mode and 5 µL for the negative mode. The liquid chromatography gradient used a 0.6 mL/min linear velocity flow rate. The gradient started at 15% B, ramped to 30% at 2 min, 48% at 2.5 min, 82% at 11 min, 99% at 11.5 min and kept at 99% B until 12 min before ramping down to 15% B at 12.1 min which was kept isocratic until 15 min to equilibrate the column. The total run time was 15 min. After every ten mouse samples, one pooled plasma QC sample was analyzed.

Targeted signal extraction and data generation: Raw LC-MS data files were converted to the ABF format and analyzed by MS-DIAL v3.0. Lipids were identified by MS/MS matching in LipidBLAST in addition to a target list of validated lipids that were routinely detected in mouse blood samples at the West Coast Metabolomics Center over the past seven years. In this database, annotated lipids are associated with retention time, adducts, m/z value and InChI keys, verified by accurate mass, isotope ratio, retention time and MS/MS spectra matching to either commercial lipid standards or to the LipidBlast library.
